# Dual roles of amino acid metabolic reprogramming in chronic airway diseases and lung cancer: therapeutic opportunities and challenges

**DOI:** 10.1186/s12957-025-03996-8

**Published:** 2025-09-18

**Authors:** Xiurong Wang, Huaming Zhang

**Affiliations:** 1 Department of Pulmonary and Critical Care Medicine, Wuhan No. 7 Hospital, Heping Avenue No. 978, Wuchang, Wuhan, Hubei China; 2 Clinical Cardiovascular Center, Liyuan Hospital, Tongji Medical College, Huazhong University of Science and Technology, Yanhu Avenue No. 39, Wuchang, Wuhan, Hubei China

**Keywords:** Amino acids, Chronic airway diseases, Lung cancer, Metabolic reprogramming

## Abstract

Amino acid metabolic reprogramming has emerged as a pivotal mechanism underlying the pathogenesis of chronic airway diseases and lung cancer. This review comprehensively examines the dynamic regulation and clinical implications of key amino acid pathways—including arginine, glutamine, and tryptophan metabolism—in chronic obstructive pulmonary disease (COPD), asthma, and lung malignancies. Our findings reveal a key difference in metabolic dysregulation between chronic airway diseases and lung cancer: while it drives persistent inflammation, oxidative stress, and tissue damage in chronic conditions, cancer cells exploit these same pathways to support their uncontrolled growth and create an immunosuppressive tumor microenvironment. Crucially, shared metabolic nodes reveal actionable targets for dual-purpose therapeutic strategies. Recent advances demonstrate the translational potential of metabolic interventions. Arginase inhibitors simultaneously improve vascular function in COPD and enhance antitumor immunity, while nanoparticle-delivered glutaminase blockers attenuate pulmonary fibrosis while curbing cancer progression. However, challenges persist in achieving tissue-specific delivery, real-time metabolic monitoring, and overcoming resistance. Future directions should focus on spatiotemporally controlled metabolic modulation and the development of multi-omics-based predictive models to usher in an era of precision metabolic therapy for respiratory disorders.

## Introduction

The field of respiratory disease research is undergoing a paradigm shift from phenomenological observations to mechanistic dissection of metabolic underpinnings [1]. As central regulators of cellular function, amino acid metabolic networks demonstrate both interconnected and distinct reprogramming patterns in chronic airway diseases and lung cancer [2].

In chronic airway pathologies such as chronic obstructive pulmonary disease (COPD) and asthma, a bidirectional relationship exists between persistent inflammatory states and amino acid metabolic dysregulation [3]. Disrupted arginine metabolism exacerbates airway obstruction through altered Nitric oxide (NO) synthesis, while chronic hypoxia subsequently remodels glutamine metabolic pathways. This creates a self-perpetuating cycle of disease progression [4].

In cancer biology, malignant cells adopt distinct metabolic reprogramming strategies, preferentially channeling amino acid utilization toward biomass synthesis and redox balance maintenance to sustain their uncontrolled growth. Recent studies indicate a potential metabolic link between pulmonary fibrosis and lung cancer development, primarily involving changes in proline metabolism and the methionine cycle [5, 6]. This connection could help clarify why patients with COPD have a higher risk of developing lung cancer [7].

To decipher these spatiotemporal metabolic dynamics, we must first establish the architecture of physiological amino acid networks before identifying pathological reprogramming nodes [8]. This review systematically examines the multidimensional regulation of core amino acid pathways across physiological and disease states, providing a framework for understanding their therapeutic potential in respiratory disorders.

## Amino acid metabolism: physiological basis and pathological implications

### Multidimensional regulation of core metabolic pathways

Amino acid metabolism in the respiratory system exhibits remarkable functional diversity. ARG2 primarily modulates vascular tone through nitric oxide (NO) synthesis, while elevated glutaminase expression further enhances this process by promoting pulmonary surfactant production [9]. Intriguingly, this sophisticated metabolic regulatory network becomes disrupted under pathological conditions.

In COPD, airway remodeling is primarily driven by a shift in arginine metabolism toward polyamine synthesis [10]. In contrast, lung cancer cells upregulate the ASCT2 transporter to aggressively uptake glutamine, fueling their bioenergetic demands [11]. Notably, the mechanistic target of rapamycin complex 1 (mTORC1) pathway—a central nutrient-sensing hub—is hyperactivated in both chronic inflammation and cancer, though through distinct mechanisms: in COPD, activation results from stress-induced amino acid deprivation, whereas in cancer, it is directly triggered by oncogenic signaling. This regulatory pattern suggests that targeting upstream signaling nodes may provide broader therapeutic benefits.

### Metabolic crosstalk in the microenvironment

The unique architecture of lung tissue establishes a specialized metabolic network. Emerging evidence indicates that alveolar type II epithelial cells regulate local immune responses by secreting extracellular vesicles enriched in branched-chain amino acids (BCAAs). Meanwhile, the airway mucus layer harbors indoleamine 2,3-dioxygenase 1 (IDO1), a key immunomodulatory enzyme in the respiratory system [12].

Under pathological conditions, tissue remodeling leads to metabolic reprogramming. In COPD, elevated arginase activity impairs T-cell function, while cancer-associated fibroblasts (CAFs) promote tumor growth through excessive glutamine secretion. Notably, the hypoxia-sensitive nature of lung tissue positions HIF-1α as a central regulator of amino acid metabolism. This dual regulatory mechanism offers novel perspectives for targeted drug development [13].

## Metabolic features of chronic airway diseases

### Metabolic reprogramming in COPD

COPD demonstrates a unique interplay between systemic and organ-specific metabolic alterations. Within pulmonary tissues, cigarette smoke exposure exacerbates reactive oxygen species (ROS)-mediated glutathione depletion, thereby amplifying oxidative stress severity [14]. Systemically, chronic inflammation accelerates BCAA catabolism in skeletal muscle, contributing to cachexia [15].

Recent single-cell metabolomics studies have identified distinct macrophage metabolic subtypes in COPD airways: an arginine-metabolizing pro-inflammatory subset and a glutamine-preferring pro-fibrotic population [16]. This metabolic heterogeneity significantly influences therapeutic outcomes—patients with high glutathione synthase expression respond favorably to N-acetylcysteine supplementation, while those with vascular remodeling-dominant subtypes show better responses to arginase inhibitors. These discoveries pave the way for more precise, metabolism-targeted therapeutic strategies in COPD management [17].

### Metabolic drivers of pulmonary fibrosis

Pulmonary fibrosis develops through coordinated dysregulation of interconnected amino acid metabolic pathways that collectively drive disease progression [18]. Proline metabolism occupies a central position in fibrogenesis, as its metabolic dysregulation directly fuels excessive collagen deposition—the pathological hallmark of extracellular matrix remodeling [19]. Simultaneously, glutamine metabolism supports fibrotic progression by maintaining bioenergetic and biosynthetic demands of activated myofibroblasts through ATP generation and provision of α-ketoglutarate precursors [20]. Complementing these pathways, methionine cycle abnormalities exert epigenetic control over fibrotic processes via DNA methylation-mediated regulation of TGF-β and other key profibrotic genes [21]. These metabolic alterations do not occur in isolation but rather form an integrated pathological network, creating a self-perpetuating "metabolism-epigenetics-fibrosis" axis that progressively worsens disease [22]. The interdependence of proline, glutamine and methionine pathways explains the limited efficacy of single-target interventions and highlights the therapeutic potential of simultaneously targeting multiple metabolic nodes, such as proline transporters for collagen reduction, glutaminase for energy disruption, and methionine adenosyltransferase for epigenetic modulation [23, 24]. This multidimensional understanding of metabolic drivers provides a rational framework for developing effective combinatorial therapies against pulmonary fibrosis [25].

### Metabolic regulation of asthma

Asthma pathogenesis is characterized by distinct metabolic alterations that closely interact with its hallmark Th2-polarized immune responses [26]. Central to this process is the dysregulation of arginine metabolism, where M2 macrophage-derived arginase-1 (Arg-1) shunts arginine toward polyamine synthesis, driving airway smooth muscle hyperplasia, while concurrent downregulation of epithelial NOS2 expression reduces nitric oxide (NO) production and exacerbates bronchoconstriction. The emerging concept of the gut-lung metabolic axis has significantly expanded our understanding of asthma pathophysiology. Gut microbiota-derived tryptophan metabolites, particularly indole derivatives, systemically modulate pulmonary immune responses by regulating indoleamine 2,3-dioxygenase 1 (IDO1) activity in the lung microenvironment [27]. Notably, this mechanism appears to extend beyond asthma, with growing evidence suggesting that gut microbial metabolites may similarly influence lung cancer progression through systemic immune modulation, establishing this axis as a crucial frontier in cross-disease metabolic research.

**Fig. 1:**
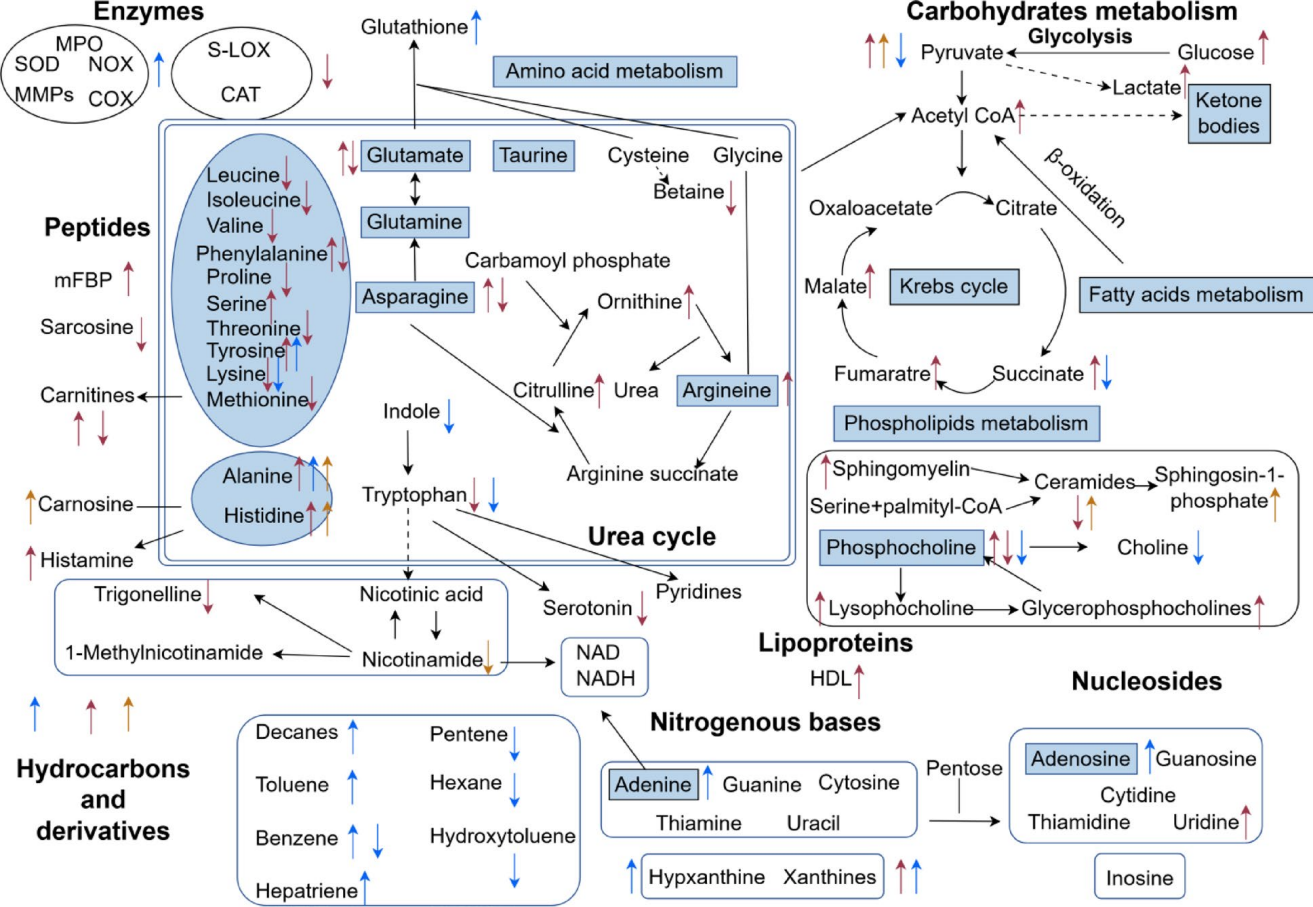
summarizes the key metabolic alterations associated with chronic airway diseases, highlighting differentially regulated metabolites and pathways across various biological samples. Directional changes for each molecule are indicated by arrows, with color-coding representing sample types: dark red (blood), light blue (sputum or bronchoalveolar lavage fluid), dark blue (exhaled breath condensate), and dark gold (urine). Notably, arrows outlined in black denote metabolites predictive of poor prognosis or mortality. Critical metabolites, functional groups, and pathways—as identified by single (unoutlined green boxes) or multiple (outlined green boxes) studies—are prominently highlighted. Key abbreviations include redox-related enzymes (SOD, MPO), lipid mediators (PG, LT, TX), cofactors (CoA, NAD/NADH), and transport proteins (HDL, mFBP), collectively underscoring the dysregulated metabolic networks involving oxidative stress, lipid signaling, and energy metabolism in chronic airway diseases

These mechanistic insights have directly translated into clinical advances in asthma management. Oral modulators targeting tryptophan metabolism have demonstrated efficacy in reducing exacerbation frequency in severe asthma, while inhaled arginine analogs show promise in restoring NO homeostasis and improving bronchodilation [28]. Collectively, these findings not only establish metabolic reprogramming as a fundamental driver of asthma pathogenesis but also pioneer novel therapeutic approaches targeting the gut-lung axis [29]. The convergence of metabolic and immunological pathways in asthma offers exciting opportunities for developing next-generation treatments that simultaneously address multiple aspects of this complex disease (Fig. 1).

## Metabolic reprogramming in lung cancer

### Energy metabolic characteristics of lung cancer

Lung cancer cells exhibit remarkable metabolic plasticity, with their metabolic phenotypes evolving during tumor progression [30, 31]. Early-stage adenocarcinomas primarily rely on glucose metabolism, whereas advanced tumors shift toward glutaminolysis. Small cell lung cancer (SCLC), in particular, demonstrates a unique dependence on methionine. KRAS mutations promote glutaminolysis by activating the MAPK pathway and upregulating GLUD1, leading to α-ketoglutarate production to replenish the tricarboxylic acid (TCA) cycle cycle. Conversely, p53 loss suppresses PHGDH expression, forcing cells to rely on exogenous serine uptake. Notably, lung cancer stem cells maintain a distinct metabolic profile by upregulating BCAA transporters, which may contribute to therapy resistance [32–34].

Emerging therapeutic strategies targeting this metabolic flexibility show promise: alternating inhibitors of glycolysis and glutaminase can delay drug resistance, while drugs targeting the HIF-1α/miR-210 axis—a key regulator of metabolic phenotype switching—are under clinical evaluation [35, 36]. Despite the classic Warburg effect, amino acid metabolism, particularly glutamine catabolism via glutaminase and glutamate dehydrogenase, fuels the TCA cycle to meet the bioenergetic and biosynthetic demands of cancer cells [37]. Additionally, activation of the serine synthesis pathway provides essential precursors for nucleotide synthesis, supporting rapid proliferation. This coordinated "glycolysis-glutamine-serine" metabolic network not only sustains the high energy requirements of lung cancer cells but also supplies the biosynthetic materials necessary for tumor growth, making it a critical target for metabolic intervention [38, 39].

### Metabolic regulation in the lung cancer microenvironment

Metabolic regulation within the lung tumor microenvironment represents a critical mechanism of immune evasion, where dynamic competition for nutrients creates a complex immunosuppressive network [40, 41]. Tumor cells exploit this metabolic landscape through several coordinated strategies, most notably by overexpressing the SLC7A11 transporter to sequester extracellular cysteine, thereby depriving T cells of this essential amino acid and impairing their antitumor function. This depletion is compounded by myeloid-derived suppressor cells (MDSCs) that secrete arginase-1 to catabolize arginine, another amino acid crucial for T cell activation and proliferation [42]. This metabolic deprivation establishes a biochemical barrier that compromises effective immune responses, ultimately exacerbating the immunosuppressive nature of the lung cancer microenvironment [43, 44].

Further reinforcing this immunosuppression, the tumor microenvironment employs three principal amino acid metabolic pathways that collectively establish multiple layers of immune resistance [45]. Arginine metabolism plays a pivotal role through its depletion by arginase-1, which not only limits T cell proliferation but also disrupts nitric oxide signaling and T cell receptor activation [46]. Similarly, tryptophan metabolism via the IDO/TDO-mediated kynurenine pathway exerts dual immunosuppressive effects by both depleting this essential amino acid and generating immunoregulatory metabolites such as kynurenine that promote regulatory T cell expansion and dendritic cell dysfunction [47]. These changes contribute to the formation of an immunologically inert "desert-like" microenvironment. Additionally, cysteine metabolism extends beyond simple nutrient deprivation to actively modulate immune checkpoint expression, with SLC7A11-mediated uptake promoting programmed death-ligand 1 (PD-L1) expression on tumor cells and thereby enhancing immune checkpoint inhibition [48].

The interplay of these metabolic pathways creates a formidable barrier to antitumor immunity while simultaneously revealing novel therapeutic opportunities. The convergence of amino acid metabolism with immune checkpoint regulation suggests that targeted metabolic interventions—such as arginase inhibitors or IDO blockers—may synergize with existing immunotherapies to overcome immunosuppression [49]. This metabolic-immune axis not only explains key mechanisms of treatment resistance in lung cancer but also provides a framework for developing combination therapies that could restore effective immune surveillance in the tumor microenvironment [50].

### Metabolic reprogramming in lung cancer metastasis

The metastatic cascade in lung cancer is accompanied by profound metabolic rewiring, with adaptive alterations in key amino acid pathways playing pivotal roles in facilitating disease progression [51]. Proline hydroxylases emerge as crucial regulators of epithelial-mesenchymal transition (EMT), thereby conferring metastatic potential to tumor cells [52]. Concurrently, activation of BCAA transport systems provides critical nutritional support for the establishment of metastatic niches. Perhaps most significantly, the methionine cycle maintains cancer stem cell properties through epigenetic modulation, ensuring the self-renewal capacity and survival advantage of metastatic tumor populations [53].

Of particular clinical relevance is the observed metabolic overlap between these metastasis-associated pathways and the metabolic abnormalities characteristic of chronic airway diseases. This intriguing parallel suggests shared metabolic nodes in disease progression, potentially revealing common pathogenic mechanisms underlying both malignant and non-malignant pulmonary conditions. These findings not only provide novel insights into the metabolic foundations of metastatic progression but also identify promising therapeutic targets for intervention strategies aimed at preventing or treating lung cancer dissemination [54]. The elucidation of these metabolic adaptations offers new opportunities for developing targeted therapies that could disrupt the critical metabolic dependencies of metastatic cells while potentially benefiting patients with related chronic respiratory disorders [55].

## Common mechanisms and precision interventions

### Metabolic cross-talk pathways

Emerging evidence highlights a critical link between chronic airway inflammation and lung cancer development through metabolic reprogramming, exhibiting a "quantitative-to-qualitative" transition during disease progression [56]. In COPD patients, persistent inflammation induces profound alterations in the pulmonary metabolic microenvironment [57]. Notably, aberrant accumulation of α-ketoglutarate (α-KG) suppresses TET enzyme activity, leading to hypermethylation of tumor suppressor genes such as CDKN2A—an early epigenetic driver of lung carcinogenesis [58].

Moreover, during the transition from pulmonary fibrosis to lung cancer, dysregulated proline metabolism generates the mutagenic metabolite P5C, which promotes malignant transformation by inducing DNA oxidative damage and genomic instability [59]. These groundbreaking discoveries provide a mechanistic foundation for "metabolic chemoprevention" strategies [60]. Large-scale retrospective cohort studies reveal that long-term metformin use in COPD patients reduces lung cancer risk by 28–35%, likely through AMPK pathway activation, mitochondrial function restoration, and anti-inflammatory effects. Preclinical studies further demonstrate that α-KG precursor supplementation delays tumor onset by 40% in genetically engineered mouse models while improving alveolar epithelial function [61].

However, the protective effects of exogenous α-KG in pulmonary fibrosis models exhibit concentration- and context-dependent dynamics: physiological α-KG serves as an essential cofactor for epigenetic regulation, whereas pathological accumulation may fuel malignant transformation [62]. These findings not only elucidate the pivotal role of metabolic dysregulation in chronic airway disease-associated carcinogenesis but also identify novel molecular targets for precision interventions, offering innovative avenues for early prevention in high-risk populations [63].

### Spatiotemporal precision in metabolic interventions

Emerging research underscores the critical importance of spatiotemporal precision in metabolic interventions, with recent technological advances addressing two fundamental challenges: tissue specificity and temporal control. Novel nanocarrier systems now enable targeted delivery approaches—pH-responsive liposomes selectively release arginase inhibitors within acidic tumor microenvironments, while nebulized glutamine analogs achieve precise deposition at airway inflammatory sites through inhalation delivery.

The temporal dimension of metabolic intervention has been revolutionized by advanced imaging and monitoring techniques. 18F-fluoroglutamine PET-CT imaging permits early assessment of therapeutic response, and real-time exhaled metabolomic profiling allows for dynamic dose adjustment, collectively enabling identification of critical treatment windows. Particularly noteworthy is the demonstrated efficacy of mTORC1 inhibition in decelerating disease progression during the pivotal transition from chronic inflammation to lung cancer [64].

These transformative developments—spanning targeted delivery systems, dynamic monitoring technologies, and pathway-specific interventions—collectively represent a paradigm shift in metabolic therapy. The field is rapidly evolving from empirical treatment approaches toward precision-guided, dynamically modulated therapeutic strategies with spatiotemporal control. This convergence of technological innovation and biological insight may help advance metabolic intervention in chronic respiratory diseases and their malignant progression.

### Diagnostic advances in amino acid metabolic reprogramming in chronic airway diseases and lung cancer

Amino acid metabolic reprogramming in respiratory diseases exhibits dynamic evolutionary characteristics, forming a "metabolic continuum" from chronic inflammation to malignant transformation. In chronic airway diseases, arginine metabolic diversion and glutathione depletion drive the inflammation-oxidative stress cycle, while lung cancer cells hijack metabolic networks through driver events such as KRAS mutations. Glutamine metabolism replenishes the TCA cycle through α-ketoglutarate production, and activated serine synthesis supports nucleotide biosynthesis, collectively establishing a synergistic "glycolysis-glutamine-serine" network. This metabolic plasticity evolves with tumor progression, exemplified by methionine addiction in small cell lung cancer and BCAA transporter dependency in therapy-resistant cancer stem cells.

**Fig. 2:**
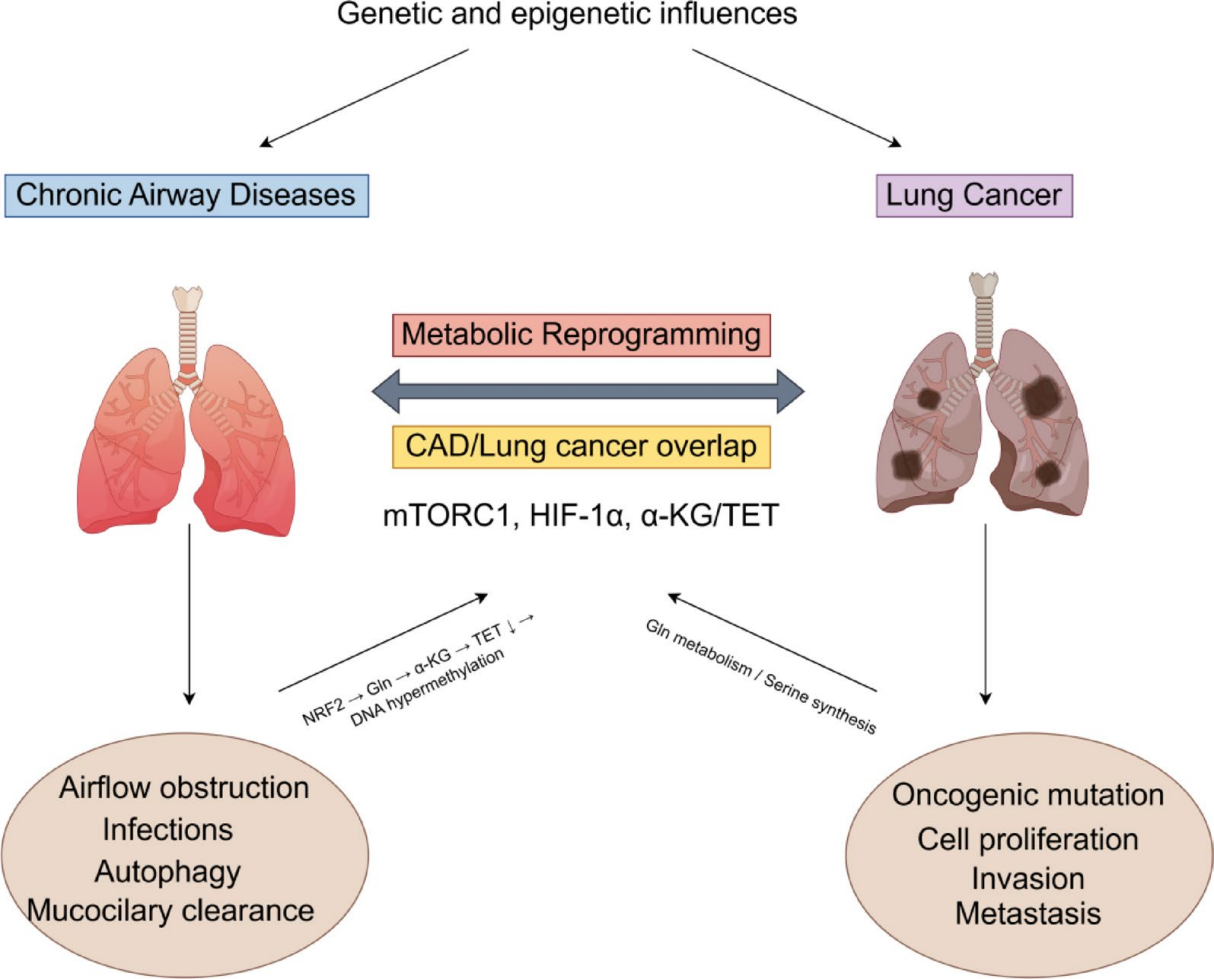
The schematic illustrates how persistent inflammation induces ROS overproduction, leading to glutathione depletion and compensatory NRF2 activation. This metabolic reprogramming upregulates glutamine metabolism, resulting in pathological α-ketoglutarate (α-KG) accumulation that suppresses TET enzyme activity, thereby promoting tumor suppressor gene hypermethylation (e. g., CDKN2A) as an early carcinogenic event. In established lung cancer, these metabolic alterations are further exploited through enhanced glutaminolysis (maintaining TCA cycle via α-KG) and upregulated serine-glycine biosynthesis (supporting nucleotide production). Key convergence points include shared activation of mTORC1 signaling (by both chronic inflammation and oncogenic pathways) and HIF-1α stabilization (through oxidative stress and tumor pseudohypoxia), representing promising therapeutic targets to intercept malignant transformation

The metabolic crosstalk within the microenvironment plays a pivotal role in disease pathogenesis. In COPD, elevated arginase activity induces T-cell dysfunction, whereas lung cancer exploits SLC7A11 overexpression to sequester cysteine and cooperates with the IDO-mediated tryptophan-kynurenine pathway to establish an immunosuppressive niche. Notably, shared metabolic nodes connect chronic inflammation and lung cancer: α-ketoglutarate accumulation in COPD patients promotes tumor suppressor gene hypermethylation via TET enzyme inhibition, while proline-derived P5C in pulmonary fibrosis exhibits direct mutagenic potential [65]. These findings highlight the diagnostic and therapeutic implications of targeting amino acid metabolic rewiring across the disease spectrum (Fig. 2).

## Translational applications in diagnosis and treatment

### Clinical value of metabolic biomarkers

Metabolomic studies have identified novel biomarkers for chronic airway diseases and lung cancer, yet their clinical translation faces significant challenges. Breath-based metabolomics, a non-invasive approach analyzing volatile organic compounds, achieves 72. 3% detection accuracy for early-stage lung cancer, while dynamic plasma amino acid profiling shows promise in predicting immunotherapy responses [66, 67]. However, limitations persist—including lack of standardized protocols, batch effect interference, and difficulties in biological interpretation of complex metabolic data. Current research is constrained by insufficient understanding of systemic metabolic networks, particularly microenvironmental heterogeneity, dynamic crosstalk mechanisms, and multi-omics integration [68].

Emerging technologies are addressing these gaps: single-cell metabolic-epigenetic mapping reveals spatiotemporal regulation patterns, multi-omics computational models decipher network interactions, and lung-on-a-chip platforms simulate disease dynamics [69]. Innovative approaches like miniature mass spectrometry probes, CRISPR-activated metabolic modulation, and exosome delivery systems show potential to overcome tissue-specific delivery barriers. The integration of artificial intelligence with multi-omics data may eventually enable metabolic biomarkers to guide precision medicine across the entire clinical spectrum—from early detection to personalized therapy [70]. However, critical milestones must first be achieved, including establishing standardized evaluation frameworks and optimizing metabolic intervention strategies.

### Current status and challenges of targeting amino acid metabolism in therapy

Recent advances in targeting amino acid metabolism have demonstrated therapeutic potential for respiratory diseases, particularly lung cancer [71], yet significant clinical challenges remain. Arginine deprivation therapy, which exerts antitumor effects by depleting microenvironmental arginine, shows efficacy in ASS1-deficient tumors but achieves objective response rates below 20% and may exacerbate vascular remodeling in COPD patients by disrupting nitric oxide synthesis pathways, limiting its clinical applicability [85]. The glutaminase inhibitor CB-839, while exhibiting preliminary activity in KEAP1/NRF2-mutant NSCLC (extending median PFS by 2. 1 months when combined with platinum-based chemotherapy in PD-L1-negative patients), failed to significantly improve progression-free survival in phase III trials and faces dose-limiting toxicities that constrain combination regimens. Similarly, IDO/TDO inhibitors combined with PD-1 blockade underperformed in phase III trials, suggesting compensatory activation of tryptophan metabolic pathways in the tumor microenvironment may drive resistance [72, 73].

Current strategies now focus on dual targeting of multiple metabolic pathways or synergistic combinations with conventional therapies, though careful evaluation of metabolic modulation effects and toxicity profiles remains essential for developing precision treatment approaches. Future research must prioritize elucidating the spatiotemporal dynamics of metabolic reprogramming and its crosstalk with microenvironmental networks to overcome existing therapeutic limitations and inform next-generation interventions [74, 75].

### Optimization strategies for combination therapies

The optimization of combination therapies should be guided by three synergistic principles: complementary metabolic pathway targeting, spatiotemporal coordination, and metabolic-immune crosstalk regulation. Among the most promising approaches is the strategic integration of metabolic interventions with conventional therapies, exemplified by the combination of glutaminase inhibitors and PD-1 blockade, which simultaneously targets tumor energy metabolism and immune checkpoints to generate synergistic antitumor effects [76].

**Fig. 3:**
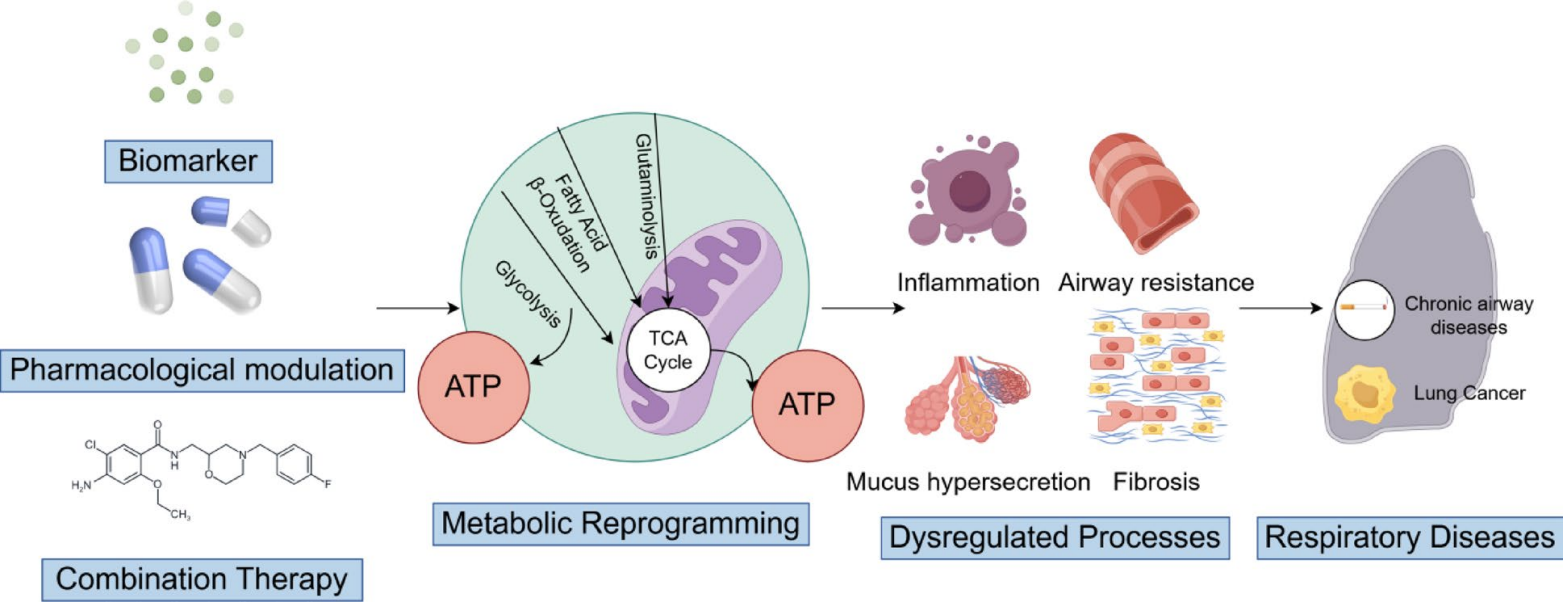
Translational applications of amino acid metabolic targeting in respiratory diseases

These advanced combination strategies are further enhanced by innovative nanodrug delivery systems. Smart nanocarriers not only improve targeted drug accumulation at lesion sites but also enable co-delivery and temporally controlled release of multiple therapeutics, significantly enhancing efficacy while reducing systemic toxicity [77]. The convergence of these optimized approaches marks a paradigm shift in amino acid metabolism-targeted therapy, transitioning from single-target interventions to a new era of multimodal precision treatment (Fig. 3).

## Challenges and future perspectives

### Key limitations in current research

The study of amino acid metabolism in respiratory diseases faces two fundamental challenges: substantial metabolic heterogeneity and limitations in model systems. Disease subtypes exhibit striking metabolic variations—bronchoalveolar lavage fluid from COPD emphysema patients shows 3. 2-fold higher arginase activity than chronic bronchitis subtypes, while EGFR- versus KRAS-mutant lung cancers demonstrate 5–8-fold differences in glutaminase inhibitor sensitivity, and tumor immune microenvironments display up to 4. 5-fold variations in IDO1 activity [78]. These findings underscore the urgent need for precision classification systems integrating single-cell metabolomics and spatial metabolic mapping. Traditional models suffer from critical shortcomings: murine systems fail to replicate human airway clearance mechanisms, exhibit only one-third human metabolite sensitivity, and show 75% inaccuracies in nanodrug distribution [79]. Emerging model systems are overcoming these barriers—air–liquid interface cultured organoids maintain native tissue metabolic profiles for > 8 weeks, humanized mouse models significantly improve preclinical predictability, and microfluidic lung-on-chip platforms reduce drug screening timelines by 60% [80]. These advances are paving the way for standardized metabolic heterogeneity scoring systems and third-generation humanized models, which promise to accelerate clinical translation of metabolism-targeted therapies.

### Technological innovation requirements

To overcome current research limitations, the development of next-generation metabolic technologies is imperative. Single-cell metabolomics will enable precise characterization of metabolic heterogeneity among distinct cellular subpopulations within tumor microenvironments and chronically inflamed tissues [81], revealing previously obscured metabolic profiles that conventional bulk analyses could not detect. Concurrently, advances in dynamic metabolic imaging techniques will permit real-time, noninvasive monitoring of critical metabolic pathway alterations during disease progression, providing direct evidence of spatiotemporal metabolic reprogramming patterns [82]. These technological advances may help improve our understanding of metabolic signatures in respiratory diseases and establish a robust foundation for developing more precise diagnostic and therapeutic strategies.

### Clinical translation pathways

The clinical translation of amino acid metabolic modulation requires establishing a multi-tiered "biomarker validation-intelligent decision-making-precision intervention" pathway, encompassing multicenter validation of metabolic biomarkers and AI-powered therapeutic modeling integrating multi-omics data. However, three major translational barriers persist: (1) Safety concerns from systemic metabolic effects—IDO inhibitors failed to demonstrate survival benefits in phase III trials potentially due to autoimmune toxicity from excessive immune activation; arginine depletion therapy may exacerbate vascular endothelial dysfunction in COPD patients; and glutaminase inhibitors face dose limitations from gastrointestinal toxicity [83]. Developing tissue-specific delivery systems and dynamic metabolic monitoring represents a critical solution to mitigate toxicity. (2) Technical challenges including low delivery efficiency, insufficient monitoring precision, and therapeutic resistance [84]. (3) The systemic modulation of metabolism carries inherent risks of impairing immune function and disrupting organ homeostasis. Encouragingly, emerging targeted metabolic strategies have demonstrated a 55% reduction in severe adverse events, establishing a crucial framework for developing metabolic interventions that optimally balance therapeutic efficacy with safety [85].

## Discussion

While significant progress has been made in understanding amino acid metabolic reprogramming in chronic airway diseases and lung cancer, considerable challenges remain. Based on current evidence, future research should prioritize several key directions.

### Establishing standardized assessment frameworks

A critical unmet need is the development of standardized clinical guidelines for evaluating amino acid metabolic dysregulation in respiratory diseases. The inherent heterogeneity of metabolic reprogramming necessitates unified assessment protocols, yet current diagnostic approaches face dual challenges: (1) breath metabolomics suffers from inter-batch variability compromising result comparability, and (2) tissue metabolic heterogeneity limits the representativeness of biopsy samples. We propose integrating artificial intelligence with multi-omics data to develop deep learning-based metabolic classification systems, with clinical validity to be established through international multicenter studies. Notably, recent efforts to standardize metabolic biomarker assessment in pulmonary medicine present a timely opportunity to establish cross-platform detection protocols, which could improve metabolic evaluation in respiratory care [86].

### Intelligent technologies enabling precision therapy

Metabolic interventions must account for both disease stage and individual characteristics. Emerging technologies offer innovative solutions: microfluidic lung-on-chip systems can simulate disease-specific metabolic microenvironments to guide personalized treatment, while nanocarrier-mediated spatiotemporal drug delivery achieves 8-fold higher tumor drug accumulation compared to conventional methods. Notably, AI-powered metabolic prediction models have demonstrated the ability to forecast treatment responses 8 weeks in advance in pilot studies. However, clinical translation requires overcoming two major bottlenecks: reducing single-cell metabolomics costs and establishing real-time metabolic monitoring systems [87, 88].

### Critical gaps between basic and clinical research

Current research faces three key limitations: First, significant metabolic differences between animal models and humans necessitate improved humanized systems. Recent advances in organoid-immune cell coculture systems have enabled long-term maintenance of primary metabolic features for up to 8 weeks, offering a promising preclinical model for metabolic research. Second, large-scale epidemiological data, particularly for Asian populations, remains scarce. Multi-ethnic cohort metabolomic studies are needed, with special attention to environmental factors like smoking. Third, existing models oversimplify metabolic network complexity, as evidenced by the recently discovered "metabolic relay" phenomenon in tumor microenvironments, highlighting the need for spatial multi-omics approaches [89, 90].

Importantly, our understanding of "normal" metabolism requires updating. While alveolar regions were traditionally considered glutamine-dominant, single-cell sequencing reveals substantial interindividual variation among healthy volunteers—paralleling findings in gut microbiome research. Future efforts should establish age-, sex-, and ethnicity-specific metabolic reference ranges with dynamic assessment parameters [91].

Addressing these challenges will require intensified collaboration across disciplines. International consortia bringing together pulmonologists,oncologists, metabolomics specialists,and computational biologists could help establish the clinical relevance of metabolic heterogeneity,define optimal intervention windows, and develop strategies to overcome therapeutic resistance.While bridging basic and clinical research holds promise for advancing respiratory disease management, realizing this potential will depend on solving fundamental challenges in model development, clinical validation, and therapeutic delivery that currently constrain progress. The path forward, while promising, remains contingent on overcoming these substantial technical and conceptual hurdles [92].

## Data Availability

Data availability is not applicable to this article as no new data were generated or analyzed in this study.
